# Making the invisible visible: Developing and evaluating an intervention to raise awareness and reduce lead exposure among children and their caregivers in rural Bangladesh

**DOI:** 10.1016/j.envres.2021.111292

**Published:** 2021-08

**Authors:** Tania Jahir, Helen O. Pitchik, Mahbubur Rahman, Jesmin Sultana, A.K.M. Shoab, Tarique Md Nurul Huda, Kendra A. Byrd, Md Saiful Islam, Farzana Yeasmin, Musa Baker, Dalia Yeasmin, Syeda Nurunnahar, Stephen P. Luby, Peter J. Winch, Jenna E. Forsyth

**Affiliations:** a Emerging Infections, Icddr,b, Dhaka, Bangladesh; b University of California, Berkeley, CA, USA; c WorldFish, 11960 Bayan Lepas, Penang, Malaysia; d School of Public Health and Community Medicine, UNSW, Sydney, Australia; e Stanford University, Stanford, CA, USA; f John Hopkins Bloomberg School of Public Health, Baltimore, MD, USA

**Keywords:** Prenatal lead exposure, Child lead exposure, Turmeric, Lead chromate, Lead-soldered cans, Nutrition, Health education and promotion, Cluster randomized trial

## Abstract

Lead exposure is harmful at any time in life, but pre-natal and early childhood exposures are particularly detrimental to cognitive development. In Bangladesh, multiple household-level lead exposures pose risks, including turmeric adulterated with lead chromate and food storage in lead-soldered cans. We developed and evaluated an intervention to reduce lead exposure among children and their caregivers in rural Bangladesh. We conducted formative research to inform theory-based behavioral recommendations. Lead exposure was one of several topics covered in the multi-component intervention focused on early child development. Community health workers (CHWs) delivered the lead component of the intervention during group sessions with pregnant women and mother-child dyads (<15 months old) in a cluster-randomized trial. We administered household surveys at baseline (control n = 301; intervention n = 320) and 9 months later at endline (control n = 279; intervention n = 239) and calculated adjusted risk and mean differences for primary outcomes. We conducted two qualitative assessments, one after 3 months and a second after 9 months, to examine the feasibility and benefits of the intervention. At endline, the prevalence of lead awareness was 52 percentage points higher in the intervention arm compared to the control (adjusted risk difference: 0.52 [95% CI 0.46 to 0.61]). Safe turmeric consumption and food storage practices were more common in the intervention versus control arm at endline, with adjusted risk differences of 0.22 [0.10 to 0.32] and 0.13 [0.00 to 0.19], respectively. Semi-structured interviews conducted with a subset of participants after the intervention revealed that the perceived benefit of reducing lead exposure was high because of the long-term negative impacts that lead can have on child cognitive development. The study demonstrates that a group-based CHW-led intervention can effectively raise awareness about and motivate lead exposure prevention behaviors in rural Bangladesh. Future efforts should combine similar awareness-raising efforts with longer-term regulatory and structural changes to systematically and sustainably reduce lead exposure.

## Highlights

• A lead exposure prevention intervention was developed in rural Bangladesh.
• Changes in knowledge and behavior were evaluated via a cluster randomized trial.
• Lead awareness and safe exposure-reducing behaviors improved post-intervention.
• One limitation of the study is that we do not know how the intervention affected blood lead levels.

## 1 Introduction

Lead exposure is harmful at any time in life, but pre-natal and early childhood exposures before age 3

are particularly detrimental to cognitive development because children's brains are undergoing rapid

development (Bellinger, 2013). Pre- and post-natal lead exposure is common in Bangladesh and other

low- and middle income countries (LMICs) (Bellinger, 2013). Among studies conducted in rural.

Bangladesh between 2007 and 2014 involving more than 1200 children and 400 pregnant women.

31−78% had elevated blood lead levels (BLLs), >5 μg/dL, compared to less than 3% in the United States

of America (CDC, 2015, 2013; Forsyth et al., 2018; Gleason et al., 2014; Mitra et al., 2012).

Not only is lead exposure more common in LMICs compared with high-income countries, but the adverse effects may also be more severe due to a higher prevalence of micronutrient deficiency (Ahamed and Siddiqui, 2007; Ettinger et al., 2009; Goyer, 1997). Poor nutritional status, especially calcium and iron deficiency, increases lead absorption and metabolism compared to fully nourished children (Goyer, 1997; Jain et al., 2005). Individuals with more lead in their bodies are also more likely to become anemic, since lead hinders heme production and red blood cell functioning, thus perpetuating a vicious cycle of undernourishment and compromised health (Hsieh et al., 2017).

In the last century, major sources of lead worldwide have included air, dust, soil, and water contaminated by gasoline, paint, and leaded pipes. In Bangladesh, however, leaded gasoline was banned in 1999, rural homes are rarely painted, and water lead levels are low (Bergkvist et al., 2010; Gleason et al., 2014; Mitra et al., 2012). Turmeric adulterated with lead chromate was identified as a primary contributor to elevated BLLs among pregnant women in several rural districts of Bangladesh, including Kishoreganj district, the focal district of this study (Forsyth et al, 2019a, 2019b, 2019a). Other sources of lead in the focal district included food stored in lead-soldered cans, along with the consumption of soil, ash, and clay, a practice known as geophagy (Abrahams et al., 2006; Al-Rmalli et al., 2010; Forsyth et al., 2018). Battery recycling and other risky occupational exposures were not found to be common in this district, although they do pose a threat elsewhere in Bangladesh (Chowdhury et al., 2021; Forsyth et al., 2018).

When designing lead exposure reduction interventions, it is important to consider not only the sources of lead but also at what scale to target change, from individuals to systems (Thomas et al., 2019). The most impactful lead exposure reduction efforts over the past 50 years have required long-term changes in policies restricting the industrial uses of lead, such as in gasoline and paint (Bellinger and Bellinger, 2006). However, policy change is neither quick nor particularly effective in LMICs like Bangladesh where the informal sector accounts for a large proportion of economic activity and environmental regulatory bodies are under-resourced and rarely enforced (Islam, 2015). Globally, banning lead in gasoline took more than four decades, and lead is still permitted in aviation fuel in many countries (Bridbord and Hanson, 2009).

During the time it takes to implement effective policies, appropriate action at different levels, including change among individuals and households could minimize the adverse effects of ongoing lead exposure. To date, individual- and household-level interventions have primarily focused on dust control to prevent exposure from leaded paint or lead acid battery recycling, but few have addressed the consumption of lead-contaminated goods (Daniell et al., 2015; Pfadenhauer et al., 2016; Yeoh et al., 2012).

Given that the three sources of lead exposure identified in rural Bangladeshi communities are related to food consumption behaviors, our objectives were to develop and evaluate a theory-based, household-level lead exposure reduction intervention. We aimed to increase awareness about and minimize consumption behaviors among caregivers and children related to the three lead sources: 1) turmeric adulterated with lead chromate, 2) food stored in lead-soldered cans, and 3) ingestion of clay, soil, or ash (geophagy). We also sought to improve diet by increasing the intake of calcium and iron-rich foods in order to decrease the absorption of lead.

## 2 Methods

### 2.1 Formative research

Between June 2015 and June 2017, an interdisciplinary team including social, environmental, and nutrition scientists carried out formative research to better understand the individual and household behaviors related to the three lead exposures (turmeric, food storage, and geophagy). The study sites were rural villages of Kishoreganj and Mymensingh districts, Bangladesh. The research team conducted semi-structured in-depth interviews and focus group discussions with 80 participants to explore behaviors related to these three exposures. Respondents were caregivers with known lead exposure (10 women with high BLLs, >5 μg/dL, and 10 with low BLLs, <2 μg/dL, as identified from another study (Forsyth et al., 2018)), as well as 40 additional caregivers from the study region whose BLLs were not measured. Focus group discussions were held with 20 of these caregivers. Individuals were first screened and then selected if they had some knowledge about or experience with at least one of the three lead exposures (e.g., owned lead-soldered cans). To probe about usage and quality aspects of turmeric and other spices, a poster with examples was shown to women during focus group discussions (Fig. S1). The team also interviewed 20 male shopkeepers selling turmeric, lead-soldered cans or clay tablets. Interviews and focus group discussions were audio recorded, transcribed, and translated. Interviews were analyzed through an inductive, thematic coding process, with attention focused on benefits and barriers to changing behaviors related to the different exposure pathways.

### 2.2 Intervention content development

Based on interviews and focus group discussion findings, we identified behavioral recommendations and developed theory-based messages to encourage actions among pregnant women and children under 2 years old that would reduce the risk of lead exposure. In order to effectively motivate participants to consider lead-reduction behaviors, we framed recommendations according to constructs from health-behavior theories: self-efficacy (a personal belief in being capable of change), identity as a nurturer (a sense of duty to keep children healthy and safe), descriptive (individual perceptions of behaviors practiced by others) and injunctive norms (individual perceptions of what others want one to do), perceived susceptibility and severity of exposure (perceptions of personal susceptibility to lead exposure and the severity of its adverse effects), and perceived benefits and barriers to action (perceptions of benefits and barriers to changing behavior to reduce the risk of lead exposure) (Cialdini et al., 1990; Rosenstock et al., 1988).

We aimed to promote different types of behaviors including changes in frequent behaviors or habits (e.g., consuming more foods rich in calcium and iron or practicing geophagy), one-time purchasing behaviors (replacing lead-soldered cans with other containers), as well as infrequent repeated behaviors (purchasing turmeric).

Before finalizing intervention content, we conducted two rounds of field tests of the messages and behavioral recommendations with a total of 32 pregnant and lactating women to assess interpretations of the messages, relevance of recommendations, effectiveness of theoretical framing, clarity of flipchart images, and duration of content delivery.

### 2.3 Intervention implementation

This lead-focused intervention was one part of a larger multiple component intervention addressing child development, nutrition, water, sanitation, and hygiene (WASH) (Pitchik et al., 2021). The sample size calculation, overall study design, and individual intervention components are described in detail elsewhere (Pitchik et al., 2021). Briefly, the intervention was delivered in 31 villages in the Katiadi and Kuliarchar sub-districts of Kishoreganj district in rural Bangladesh by community health workers (CHWs) between October 2017 and May 2018. Villages in the Katiadi and Kuliarchar subdistricts consist of 200–800 households with an average of five members per household. In any typical village in Bangladesh, the main livelihood is agriculture. Villages were included in the study if the basic demographic characteristics (e.g., household size, literacy, electricity) were within 1.5 standard deviations of the district average to minimize the risk of chance imbalance in demographic characteristics in the intervention and control villages at baseline.

This was a cluster-randomized controlled trial where each cluster was comprised of a single village. Through stratified randomization, 31 villages were randomly assigned to a control arm (n = 15), an intervention arm that consisted of group sessions only (n = 8), or an intervention arm that consisted of a combination of group sessions and home visits (n = 8). The lead intervention was delivered in group sessions to all participants, as such we compared the 16 intervention villages to the 15 control villages in all analyses. The allocation ratio of the intervention to control villages was 10:8 in Katiadi and 6:7 in Kuliarchar sub-districts. Eligible participants were women living in the selected villages that were in their 2nd or 3rd trimester of pregnancy, or whose youngest child was less than 15 months of age. Upon enrollment and prior to data collection, we obtained written informed consent from adult participants. The study protocol was reviewed and approved by the Institutional Review Boards at the University of California, Davis and icddr,b.

#### 2.3.1 Quantitative data collection

Participants learned of their intervention assignment after the baseline survey and data collectors were blind to participants’ assignments. At baseline we assessed demographic characteristics, and at both baseline and endline we assessed participants knowledge and behaviors related to lead exposure prevention. Knowledge of lead was defined as basic awareness of lead as an environmental toxin and measured with a question about if the participant had ever heard of lead. Further questions were asked to ascertain knowledge of the adverse effects of lead exposure, common sources of exposure, and ways to reduce exposure. Turmeric purchasing patterns were determined by asking the participants what type of turmeric they purchased, with visual prompts indicating to differentiate polished and unpolished turmeric. Food storage was measured by observation of the containers that participants used to store foods in the household. Geophagy was estimated by asking about the consumption of soil, ash, and clay during pregnancy. To determine consumption of calcium- and iron-rich foods, we asked caregivers to recall all the foods eaten in the last 24 h by both the caregiver and the focal child. We then grouped food recall responses into one of eight calcium- and iron-rich food groups that we defined based on known calcium and iron content. Nine months later, at intervention endline in May 2018, we re-administered the same survey.

#### 2.3.2 Quantitative analysis

Sample size calculations were based on the primary child development outcome from the multi-component intervention and are described elsewhere (Pitchik et al., 2021). We estimated differences in lead-related outcomes between intervention and control arms at endline. We estimated risk differences for binary outcomes (lead knowledge and prevalence of safe and risky turmeric and food storage behaviors), and mean differences for continuous outcomes (number of calcium- and iron-rich food groups consumed). In all analyses, we adjusted for relevant covariates such as child age, child sex, maternal education, housing construction materials, household assets, and the outcome of interest measured at baseline. For each outcome, covariates were prescreened using a likelihood ratio test, and those with p < 0.20 were included in adjusted models. We used the parametric g-formula (R package: riskCommunicator) to estimate differences in outcomes between the intervention and control arm (Ahern et al., 2009; Jessica A. Grembi and McQuade, Elizabeth T. Rogawski, 2020; Pitchik et al., 2021). This approach follows four steps: 1) fit a regression model, 2) estimate counterfactuals, 3) estimate marginal differences, and 4) construct 95% confidence intervals by cluster bootstrapping resampling with 1000 replicates. Residuals were normally distributed, and variables did not need to be transformed. Analyses were intention to treat, meaning that they were conducted according to the randomized intervention arm at enrollment, regardless of session attendance. The trial is registered in ISRCTN with primary outcomes specified related to child development (Family Care Indicators; ISRCTN16001234).

#### 2.3.3 Qualitative data collection

We conducted two qualitative assessments during the 9-month study period: one at 3 months and the second at 9 months to explore the feasibility and the acceptability of the behavioral recommendations for each group of respondents and to solicit feedback regarding the session content and opportunities to improve content and delivery. In total, the study team carried out 11 focus group discussions with participants and 5 with CHWs, as well as 31 interviews with participants and 4 with supervisors of CHWs.

#### 2.3.4 Qualitative analysis

Trained qualitative researchers conducted focus group discussions and in-depth interviews in Bengali. On average, interviews were 45 min and focus groups 90 min. Interviews were conducted one-on-one and in the focus groups, two researchers were involved: one as facilitator, and other as notetaker and co-facilitator. Data collection followed semi-structured guides that were developed based on the study objectives and research questions.

All focus groups, and interviews were audio-recorded then transcribed in Bengali. The research team generated themes and codes (deductive) prior to the data collection and coded the transcripts manually. We did not use any software. While coding, new and emerging (inductive) codes were also included. All coded data were compiled according to themes. Coded portions were translated into English. Thematic content analysis was used to describe findings.

## 3 Results

### 3.1 Formative research

In rural areas of Bangladesh, including our focal district, residents live in compounds where 2 or more families live inside a single boundary and share a common courtyard, cooking space, toilet, and water source (tube well). Within a compound, one woman was responsible for cooking for immediate and extended family members, usually once per day. Women described turmeric as an essential spice, a critical addition to curries with meat or fish to enhance the color of the dish. The amount of turmeric added should be “just right” to obtain a nice yellow color, but not so much to create a bitter taste. Turmeric quality was described as relating to a woman's perceived self-worth because of its impact on the look and taste of the food she prepared. However, men were responsible for purchasing turmeric. All respondents were familiar with the general concept of food adulteration, locally known as *vejal*, but were unaware of the specific issue of adulteration with lead chromate to enhance the yellow color of turmeric. Instead, women were concerned about turmeric powder adulterated with flour, sawdust, or other substances that would dilute the vibrant yellow color and require an individual to add more turmeric to curries to achieve the desired color. Although dried turmeric roots were considered purer by the participants than turmeric powder, they were less available at small local bazaars in rural villages. Because of this, some women formed a co-op where one individual procured and ground dried turmeric roots for an entire compound to ensure that it was free from adulteration.

**Fig. 1:**
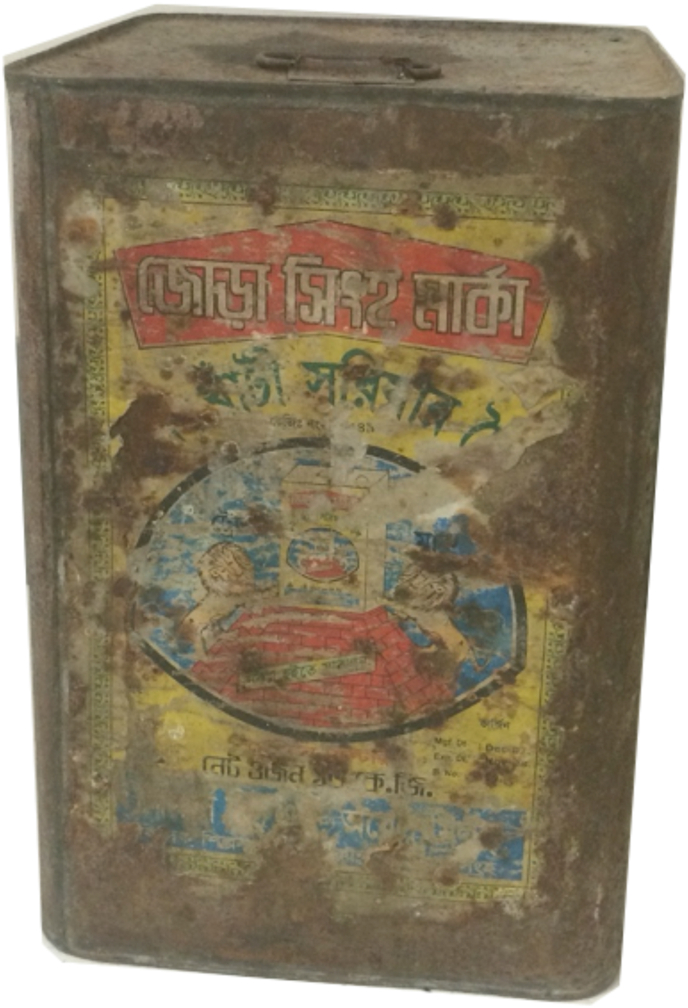
Example of a lead-soldered can. The lead is visible near the top seam of the can.

Women reported using lead-soldered cans to store dried goods: rice, biscuits, dried fish, and spices (Table S1, Fig. 1, Fig. S2). Most respondents with lead-soldered cans had multiple cans that were purchased by the male head of household. Some cans were purchased with lead solder and other cans were repaired with lead solder after 5–12 years. Lead-free alternative storage containers like plastic containers were considered to be acceptable and were increasing in popularity and availability despite some minor disadvantages ranging from strong plastic smells to less efficient pest deterrence. Respondents mentioned door-to-door salesmen were selling high-quality, affordable, plastic containers, making them the easiest to procure.

Women reported that geophagy was most common during pregnancy and soil and clay consumption included: 1) soil and ash found around earthen stoves (*sikkamati* and *ghoborer chai,* Figs. S3) and 2) pieces of unused or broken clay pots or toys, and 3) fired clay tablets specifically formulated for consumption during pregnancy (*tirhi)* (Table S2). The primary reasons to consume soil, ash or clay were cravings triggered by the “sweet smell and taste of the earth.” The frequency of consumption varied from daily to 1–2 times throughout pregnancy. All respondents knew someone who had consumed clay pots, pieces of clay toys, soil and ash found around earthen stoves though respondents mentioned that the practice was becoming increasingly uncommon. The practice was stigmatized and perceived to be unhealthy by family members and friends in the community.

### 3.2 Intervention content development and implementation

The theory-based messages and behavioral recommendations were based on findings from formative research and field testing (Table 1, additional details provided in Sections 2, 3 of the Supplementary Material). Although our intervention targeted caregivers, the formative research revealed levers for change at different levels and the potential benefit of targeting government officials and business people in subsequent interventions (Table S3).

**Table 1:** Behavioral recommendations and theoretical constructs for prevention of lead exposure at household level.

Category	Recommendations	Theoretical constructs(Funnell and Rogers, 2011)	Example messages
Turmeric	Buy raw turmeric root or unpolished dried turmeric root, and paste or grind it oneself, instead of buying powdered turmeric or polished turmeric root.	1. Increase self-efficacy to cook with lead-free raw or unpolished turmeric via demonstrations 2. Increase perceived benefits of unpolished turmeric 3. Reduce perceived barriers of switching to unpolished turmeric	“Some grinders cheat you by adding chickpea flour to powdered turmeric. Since unpolished turmeric is the purest, it will make the most colorful curries.” (Perceived benefits) “Unpolished dried turmeric may be less available than polished and powdered turmeric, but if you coordinate with women in this group, one person could purchase and grind turmeric for everyone in the group.” (Perceived barriers)
Food storage	Use high-quality, food-grade plastic container for storing food instead of lead-soldered cans.	1. Increase perceived benefits of alternative lead-free containers 2. Alter descriptive norms	“High-quality, food-grade plastic containers are better than lead-soldered metal cans because they are more available, more affordable and more durable (no rust and do not need to be repaired).” (Perceived benefits) “Everyone uses plastic containers these days, you can even get them from door-to-door salesmen.” (Perceived benefits, descriptive norms)
Geophagy	Smell lemon leaves or eat ginger when feeling nauseous.	1. Increase perceived benefits of alternative behaviors 2. Alter descriptive norms	“We are humans so we should consume food and not soil. Lemon leaves and ginger make you better but soil and clay harm you.” (Perceived benefits) “Pregnant women are not consuming as much soil or clay because they understand how harmful it is.” (Descriptive norms)
Nutrition	Consume at least 5 calcium- and iron-rich food groups in a week to reduce lead absorption in the body.	1. Increase perceived benefit of diverse food consumption	“Consume as many calcium- and iron-rich foods as you can (at least 5) because these foods will strengthen your body to fight against lead.” (Perceived benefits)

To introduce the concept of lead exposure, we drew analogies with arsenic, an invisible, yet well-known toxin. For each specific recommendation, we created flipcharts with pictures to convey “what to avoid” “why to avoid” and “what to do instead.” We communicated basic information about lead and its adverse effects, followed by specific recommendations to minimize the burden of exposure related to the focal sources: 1) avoid purchasing risky turmeric (loose powdered or polished dried turmeric roots) and instead purchase safe turmeric (unpolished roots), 2) avoid storing food in risky containers (lead-soldered cans) and instead choose safe containers (glass, high-quality food-grade plastic, or lead-free metal containers), 3) avoid consuming soil, clay or ash to combat nausea or pregnancy cravings and instead try ginger or lemon, and 4) increase the intake of calcium- and iron-rich food groups such as leafy greens, legumes and pulses, nuts and seeds, dairy, eggs, and meat. From field tests, we found that mothers were only able to differentiate polished and unpolished turmeric roots after seeing images presented. To further improve understanding, we decided that CHWs would show participants physical examples of polished turmeric as well as lead-soldered cans. Participants were encouraged to interact with and touch those objects.

Based on feedback from field tests, we decided to deliver the entire lead topic in a single group session. This component took 25–30 min. Each intervention session started with child stimulation components followed by nutrition and/or WASH. CHWs delivered the lead content in the first 30-min of the group session, with reminders during three follow-up group sessions held 6, 16, and 20 weeks after the initial lead session. Each of the reminder sessions took 5–10 min for the lead component. In the review sessions, group affirmations were used to praise participants who exhibited awareness and behaviors to reduce lead exposure.

### 3.3 Intervention evaluation

#### 3.3.1 Baseline characteristics

In total, 621 women were enrolled in the pilot trial at baseline, with 301 in the control and 320 in the intervention arm. In each village there were an average of 20 pregnant women and caregivers of children <15 months of age. At endline, 568 women were followed-up with lead-related questions; 279 in the control arm and 289 in the intervention arm. Loss to follow-up was not significantly different between study arms. The full trial profile and loss to follow-up data are presented in detail elsewhere (Pitchik et al., 2021). Baseline socioeconomic, demographic, and exposure characteristics were similar between the intervention and control arms (Table 2).

**Table 2:** Baseline characteristics of the target mother, child, and household. Data are n (%) or mean (SD).

	Control (N = 301)	Intervention^a^ (N = 320)
**Maternal characteristics**		
Age in years	25.0 (5.6)	25.0 (6.1)
Years of education	6.2 (3.4)	6.0 (3.3)
Pregnant	61 (20%)	64 (20%)
Achieved minimum dietary diversity^b^	182 (60%)	189 (59%)
**Child characteristics (Control = 240, Intervention = 254)**		
Age in months	6.6 (3.9)	6.6 (4.0)
Achieved minimum dietary diversity^c^ (n = 272)	45 (35%)	47 (33%)
Middle upper arm circumference (MUAC)^d^ < 12.5 (n = 296)	11 (8)	13 (8.5)
**Household characteristics**		
Years of education, household head	8 (18)	6.7 (14)
Household size	5.2 (2.2)	5.3 (2.3)
Has electricity	243 (81%)	289 (90%)
Has radio	4 (1%)	6 (2%)
Has television (either color or black & white)	70 (23%)	87 (27%)
Has cement floor	65 (22%)	54 (17%)
Has brick wall	74 (25%)	53 (17%)
**Knowledge**		
Aware of lead	67 (22%)	96 (30%)
Mentioned adverse effect on cognitive development	0 (0)	0 (0)
Know how to avoid harmful effects of lead	1 (2%)	0 (0)
Pregnant women should not eat soil or clay	–	–
Buy safe turmeric (self-grown, raw or dried unpolished)	–	–
Store food in safe containers (plastic, clay or metal with no lead solder)	–	–
**Behaviors**		
Turmeric^e^^,^^f^		
Daily turmeric consumption, teaspoons per person per day	0.65 (0.37)	0.61 (0.32)
Safe turmeric (self-grown, raw or dried unpolished)	137 (46%)	130 (41%)
Risky turmeric (powdered or dried polished)	177 (59%)	198 (62%)
Food storage^f^		
Safe containers (plastic, metal or clay)	59 (20%)	91 (29%)
Risky containers (lead-soldered can)	10 (3.3)	15 (4.7)
Geophagy		
Consumed soil or clay during pregnancy	14 (5%)	19 (6%)
Number of calcium and iron-rich food groups consumed^g^		
Maternal	2.0 (0.9)	1.9 (0.9)
Child	0.8 (1.0)	0.8 (1.0)

a Combined intervention arms (group and combined) from the integrated intervention(Pitchik et al., 2021).

b Mother reported eating 5 or more food groups in the last 24 h, out of the following groups: grains, legumes, nuts and seeds, dairy products, animal flesh foods, eggs, vitamin A rich fruits and vegetables, other vitamin A rich fruits and vegetables, other vegetables, other fruits(Pitchik et al., 2021).

c Children >6 months reported eating 5 or more food groups in the last 24 h, out of the following groups: breast milk, grains, legumes, dairy products, animal flesh foods, eggs, vitamin A rich fruits and vegetables, other fruits and vegetables (n = 272, control = 129, intervention = 143)(Pitchik et al., 2021).

d Only index children >6 months of age at baseline are included (n = 296, control = 144, intervention = 152).

e 1 teaspoon is equal to roughly 2–3.5 g turmeric depending on how finely ground and how tightly packed the turmeric powder is.

f Presenting results for both “safe” and “risky” behaviors since a single household can be engaging in both simultaneously.

g Number of calcium and iron-rich food groups eaten in the last 24 h, out of the following 8 groups: i) leafy green vegetables, ii) organ meat, iii) flesh meat, iv) eggs, v) fish and seafood, vi) grains, legumes, nuts and seeds, vii) dairy products (other than milk) and viii) milk.

#### 3.3.2 Quantitative assessment: intervention impacts on knowledge and behavior

At endline, the adjusted risk difference for lead knowledge was 0.52 [95% CI 0.46 to 0.61], which indicated that the prevalence of lead awareness was 52 percentage points higher in the intervention arm compared to the control arm (Table 3). Of those familiar with lead, awareness that lead impairs child development was 48 percentage points higher intervention arm compared to the control arm (0.48 [0.41 to 0.61]). Similarly, knowledge about ways to avoid harm from lead was higher in the intervention arm compared to the control arm (0.54 [0.46 to 0.66]).

**Table 3:** Comparison between the control and intervention arms related to lead risk reduction knowledge and behaviors at endline.

	Control Freq (%) n = 279	Intervention Freq (%) n = 289	Adjusted^a^ Risk Difference (95% CI)
**Knowledge**			
Aware of lead	68 (24%)	213 (74%)	0.52 (0.42, 0.61)
Mentioned adverse effect on cognitive development	2 (1%)	137 (47%)	0.48 (0.41, 0.61)
Know how to avoid harmful effects of lead	8(3%)	151 (52%)	0.54 (0.46, 0.66)
Pregnant women should not eat soil or clay	0 (0%)	25 (9%)	0.16 (0.11, 0.43)
Buy safe (unpolished) turmeric instead of risky (powdered or polished)	2 (1%)	56 (19%)	0.28 (0.19, 0.53)
Store food in good quality plastic containers or metal can with no lead solder	6 (2%)	130 (45%)	0.58 (0.43, 0.74)
**Behaviors**			
Turmeric^b^			
Safe turmeric (self-grown, raw or dried unpolished)	85 (31%)	133 (46%)	0.22 (0.10, 0.32)
Risky turmeric (powdered or dried polished)	197 (71%)	157 (54%)	−0.18 (−0.29, −0.08)
Food storage^b^			
Safe containers (plastic, metal or clay)	227 (81%)	274 (95%)	0.13 (0.00, 0.19)
Risky containers (lead-soldered can)	14 (5%)	10 (4%)	−0.02 (−0.06, 0.03)
			Adjusted^c^ Mean Difference (95%CI)
Number of calcium and iron-rich food groups consumed^c^			
Maternal	2.0 (1.0)	2.1 (0.9)	0.10 (−0.05, 0.25)
Child	1.7(1.1)	2.1(1.1)	0.37 (0.12, 0.6)

a We adjusted for potential covariates: child age, child sex, maternal education, housing construction materials and household assets.

b Presenting results for both “safe” and “risky” behaviors since a single household can be engaging in both simultaneously.

c Number of calcium and iron-rich food groups eaten in the last 24 h, out of the following 8 groups: i) leafy green vegetables, ii) organ meat, iii) flesh meat, iv) eggs, v) fish and seafood, vi) grains, legumes, nuts and seeds, vii) dairy products (other than milk) and viii) milk.

More intervention than control respondents reported positive changes in behavior for all exposure pathways assessed at endline (Table 3). Prevalence of safe turmeric consumption was significantly higher in the intervention versus control arm at endline (adjusted risk difference: 0.22 [0.10 to 0.32]). Meanwhile, risky turmeric consumption was lower in the intervention versus control arm (−0.18 [-0.29 to −0.08]). There was a modest increase in the number of calcium- and iron-rich food groups consumed by children in the intervention versus control arm at endline (adjusted mean difference: 0.37 [0.12 to 0.6]. Mothers in the intervention arm also reported consuming more calcium- and iron-rich food groups than those in the control group (adjusted mean difference: 0.1 [-0.05 to 0.25], but the difference was less than that of children. Due to the low prevalence of geophagy at baseline (<5%) and the limited number of pregnant respondents (20%), geophagic practices were not re-assessed at endline (Tables S4 and S5).

In addition to self-reported behaviors, the field team confirmed reported food storage behavior with observations. The prevalence of safe food storage was higher in the intervention versus control arm at endline (adjusted risk difference: 0.13 [0.00 to 0.19]). Risky food storage, however, was similarly low, 5% or less, in both arms.

#### 3.3.3 Qualitative assessment: perceptions of the intervention implementation and content

Among all of the topics included in the integrated intervention, participants mentioned that lead-related information was the most interesting because it was a high-risk exposure that participants were not familiar with. As a result, both the respondents and CHWs reported sharing information about lead with relatives and neighbors. Participants reported that they found the comparison of lead to arsenic to be salient. For the most part, perceived barriers to changing behaviors were manageable because the behavioral recommendations aligned with participants’ aspirations and they were motivated to make the changes. As mothers, they mentioned that they would do anything to avoid the long-term adverse impacts associated with child lead exposure and do their best to ensure that their children have a happy productive life. Participants did report difficulties associated with changing turmeric consumption: i) mothers were less concerned about child lead exposure from turmeric since their children were too young to eat spicy foods, and ii) turmeric was purchased infrequently and in large quantities by male heads of households. Mothers were not willing to discard the large amount of turmeric that had already been purchased.

Findings from these qualitative assessments informed changes to session content. For example, after learning that men predominantly purchase turmeric and lead-soldered cans, the field team convened groups of male head of households during the last two sessions (weeks 16 and 20) in order to directly communicate the relevant intervention messages and behavioral recommendations. Several women reported that these sessions convinced their husbands to purchase pure unpolished turmeric root.

## 4 Discussion

This study demonstrates that a household-based CHW-led multiple component intervention raised awareness about lead, an unknown invisible toxin, in rural Bangladesh. The intervention improved respondents' understanding of the neuro-developmental damage from childhood lead exposure, and ways to reduce household lead exposure. One of the strengths of this intervention was our iterative approach to intervention development, involving formative research and field testing to ensure that behavioral recommendations were salient. We also made sure that the behavioral recommendations were feasible, appropriate, and framed using theoretical constructs that aligned with caregivers’ motivations to raise healthy and productive children.

Although the intervention originally targeted mothers and children vulnerable to the adverse effects of lead exposure, we adapted the intervention to involve male heads of households responsible for purchasing household items to facilitate behavior change. Elsewhere, lead education interventions have targeted both women and men. A household-level intervention study conducted in the United States of America addressed lead exposure from indoor dust and targeted women because they were more likely to be responsible for cleaning (Jordan et al., 2003). Another intervention in Vietnam aimed to reduce lead exposure from battery recycling and therefore targeted men because they are more likely to be occupationally exposed (Daniell et al., 2015). We recommend that subsequent efforts to prevent and control lead exposures at the household-level consider who in the household is most capable of making the requisite changes.

The novelty of the concept of lead, combined with the threat of the adverse impacts of lead exposure, may explain the interest with which women participated in the intervention and the notable improvements in knowledge and behavior. A household-level lead prevention intervention in the US noted that women were more responsive if they had never heard about the issue before (Jordan et al., 2007). CHWs made the concept of lead itself relatable by comparing it to arsenic, a familiar invisible toxin. Since respondents were familiar with arsenic, they were able to understand the concept of lead exposure; specifically, the notion that lead could pose a threat without being seen or detected.

Since lead is usually invisible, perceived susceptibility can be low and risk communication can be difficult. Of the focal exposure pathways in this study, only the lead solder on food storage cans was visible. Even though lead could not be seen in turmeric powder, food adulteration is so common in Bangladesh that respondents did not doubt CHWs when told that lead chromate might be added to their turmeric. Respondents were not familiar with turmeric processing, so the examples of polished and unpolished turmeric roots that CHWs brought as examples helped respondents identify safe and risky turmeric when they visited bazaars.

Some respondents reported that they preferred to use up their existing turmeric stock before purchasing safer turmeric in part because they could not determine if their turmeric contained lead and also because they purchased large quantities infrequently. The intervention may have had an even more pronounced effect if respondents had been able to assess lead concentrations in their turmeric and other exposure sources. Currently, there is not a simple or inexpensive test to detect lead in turmeric and other household items. X-ray fluorescence (XRF) analyzers can be used for rapid lead screening of spices but they cost more than $15000, and are more be appropriate for governmental than individual use (Palmer et al., 2009).

Integrating interdisciplinary topics in a single intervention can be effective so long as the numerous behavioral recommendations reinforce each other and do not overwhelm respondents. This multiple component intervention covered several topics in addition to lead exposure prevention: child stimulation, maternal mental health, water, sanitation, and hygiene (WASH), and nutrition (Pitchik et al., 2021). Most other lead-focused interventions primarily focus on changing habits like cleaning, dusting, or handwashing, which align well with the traditional WASH messages. In this study, lead risk reduction behavioral recommendations were related to consumption and therefore aligned best with nutrition messages. This natural alignment enabled us to reiterate different reasons for taking the same action which may have made these recommendations more convincing and less overwhelming.

Additionally, our behavioral recommendations used theoretical framing to minimize perceived barriers and highlight salient benefits of safer alternatives. Although we utilized individual-based theoretical models to inform our framing, we also considered spheres of influence such as male heads of household who were responsible for purchasing decisions. Beyond the individual level, we addressed factors from ecological models like the challenges associated with finding unpolished turmeric roots in village bazaars and grinding them into powder on a small-scale. During intervention group sessions, we facilitated inter-household coordination to collectively procure and grind turmeric roots instead of doing each step as individuals. To better ascertain mechanisms of behavior change, future studies should measure theoretical constructs directly, including risk perception, since behavior change may be moderated by perceived risk (Grasmück and Scholz, 2005).

One limitation of the study is that, despite our best efforts, we may have missed sources of lead exposure. We specifically targeted food and food-related items because these are the sources have been identified in the study region (Forsyth et al, 2018, 2019b). However, studies conducted in Bangladesh and other LMICs have highlighted the impact of lead acid battery recycling on child lead poisoning. Due to the informal, transient nature of battery recycling, operators frequently abandon sites and move, making this a possible ongoing threat (Chowdhury et al., 2021). Although there were no known lead-polluting industries in our site like battery recycling at the time of our study, new battery recyclers may move into the area and subsequent studies should continue to re-assess the presence of both household and non-household exposure sources. Another limitation of the study is that we do not know how the intervention affected blood lead levels. Measuring baseline and endline blood lead levels would have enabled us to more easily compare the effectiveness of our intervention with other studies. A final limitation is that courtesy bias may have encouraged respondents to over-report recommended behaviors. The observed increased prevalence in safe food storage provides some reassurance that at least some of the reported improvements were genuine.

With minimal research into the effectiveness of lead education interventions in LMICs, we present evidence from interventions conducted in high income countries as a comparison. A systematic review of 13 intervention studies conducted in the United States of America and Australia found that household-level action alone may not be sufficient to produce sustained reductions in blood lead levels (Nussbaumer-Streit et al., 2016). For example, an intervention educating women about cleaning practices to reduce lead-contaminated dust exposure in painted homes only reduced blood lead levels for 6 months (Tohn et al., 2003). In order to reduce blood lead levels for longer periods of time, policies banning leaded paints and structural interventions to encapsulate lead in homes were required (Dixon et al., 2005). Such regulatory and structural changes can take years to implement. As a result, efforts to empower individuals to reduce their own risks may fill a gap in the interim, especially in lower income settings.

Consistent with the studies described above, turmeric and the other focal lead exposures in this study can be avoided with individual- and household-level action even though change is still needed at higher levels and across the turmeric supply chain in order to ensure sustainable reductions in exposure. Such changes are already underway in Bangladesh. After the news spread about lead chromate adulteration in turmeric in late September 2019, the Prime Minister announced plans to restrict the use of lead chromate (Rezaul Karim, 2019). In the same month, the Bangladesh Food Safety Authority took swift action; they penalized turmeric purveyors selling lead-containing roots, and developed a monitoring and inspection plan to ensure that adulterated turmeric is confiscated before it reaches the consumer.

## 5 Conclusion

Overall, this study demonstrates that a group-based CHW-led multiple component intervention can effectively raise awareness and change behavior to reduce risk from a previously unknown invisible toxin in rural Bangladesh. Future efforts should combine similar household- and individual-level awareness-raising efforts with regulatory action and longer-term initiatives to sustain reductions in lead exposure at different levels. Moreover, these efforts should be expanded across Bangladesh and globally to other countries like Georgia, Pakistan, Nepal, and Morocco, where turmeric and other spices have recently been found to have high lead levels (Hore et al., 2019).

## Declaration of competing interest

The authors declare that they have no known competing financial interests or personal relationships that could have appeared to influence the work reported in this paper.

## Declarations

### Availability of data and material

The datasets used during the current study are available from the corresponding author on reasonable request.

## Credit statement

Tania Jahir: Conceptualization, Investigation, Formal analysis, Data curation, Project administration, Writing – review & editing. Helen O. Pitchik: Formal analysis, Data curation, Writing – review & editing. Mahbubur Rahman: Investigation, Project administration, Writing – review & editing, Supervision. Jesmin Sultana: Investigation, Data curation, Project administration. A.K.M. Shoab: Data curation, Project administration. Tarique M.N. Huda: Investigation, Project administration, Writing – review & editing, Supervision. Kendra A. Byrd: Formal analysis, Writing – review & editing. M. Saiful Islam: Writing – review & editing. Farzana Yeasmin: Investigation, Project administration, Writing – review & editing, Supervision. Musa Baker: Investigation, Writing – review & editing. Dalia Yeasmin: Investigation, Writing – review & editing. Syeda Nurunnahar: Investigation, Writing – review & editing. Stephen P. Luby: Conceptualization, Writing – review & editing. Peter J. Winch: Conceptualization, Methodology, Writing – review & editing. Jenna E. Forsyth: Conceptualization, Methodology, Software, Formal analysis, Data curation, Writing – original draft, Visualization.
