# Supplementary material for: Making the invisible visible: Developing and evaluating an intervention to raise awareness and reduce lead exposure among children and their caregivers in rural Bangladesh
Source: Environ Res. 2021 Aug;199:111292. doi: 10.1016/j.envres.2021.111292 (PMC8261827; doi:10.1016/j.envres.2021.111292)
Supplement: Multimedia component 1 [file mmc1.docx]

**Supporting information for:**

**Title:** Making the invisible visible: developing and evaluating an intervention to raise awareness and reduce lead exposure among children and their caregivers in rural Bangladesh

Authors: Tania Jahir^1^, Helen O. Pitchik^2^, Mahbubur Rahman^1^, Jesmin Sultana^1^, AKM Shoab^1^, Tarique Md. Nurul Huda^1^, Kendra A. Byrd^3^, Md. Saiful Islam^1,4^, Farzana Yeasmin^1^, Musa Baker,^1^ Dalia Yeasmin,^1^ Syeda Nurunnahar,^1^Stephen P Luby,^5^ Peter J Winch,^6^ Jenna E. Forsyth^5^

**CORRESPONDING AUTHOR**

Jenna E. Forsyth, Stanford University, Stanford, USA, email: [jforsyth@stanford.edu](mailto:jforsyth@stanford.edu)

AUTHORS’ AFFILIATION AND ADDRESSES:

^1^Emerging Infections, icddr,b, Dhaka, Bangladesh e-mails: [tania.jahir@icddrb.org](mailto:tania.jahir@icddrb.org), [mahbubr@icddrb.org](mailto:mahbubr@icddrb.org), [jesmin.sultana@icddrb.org](mailto:jesmin.sultana@icddrb.org), akmshoab@icddrb.org, [tarique.huda@icddrb.org](mailto:tarique.huda@icddrb.org), [fyeasmin@icddrb.org](mailto:fyeasmin@icddrb.org), [musa.baker@icddrb.org](mailto:musa.baker@icddrb.org), [daliay@icddrb.org](mailto:daliay@icddrb.org), [nurunnahar@icddrb.org](mailto:nurunnahar@icddrb.org)

^2^University of California, Berkeley, California, USA, e-mail: [hpitchik@berkeley.edu](mailto:hpitchik@berkeley.edu)

^3^WorldFish, 11960 Bayan Lepas, Penang, Malaysia, e-mail: [K.Byrd@cgair.org](mailto:K.Byrd@cgair.org)

^4^School of Public Health and Community Medicine, UNSW, Sydney, Australia, e-mail: [mdsaiful.islam@unsw.edu.au](mailto:mdsaiful.islam@unsw.edu.au),

^5^Stanford University, Stanford, California, USA, e-mail: [sluby@stanford.edu](mailto:sluby@stanford.edu), [jforsyth@stanford.edu](mailto:jforsyth@stanford.edu)

^6^John Hopkins Bloomberg School of Public Health, Baltimore, Maryland, USA, e-mail: [pwinch@jhu.edu](mailto:pwinch@jhu.edu)

# **S1. Pictures and findings from formative research related to lead sources**

# **S1-1. Turmeric**


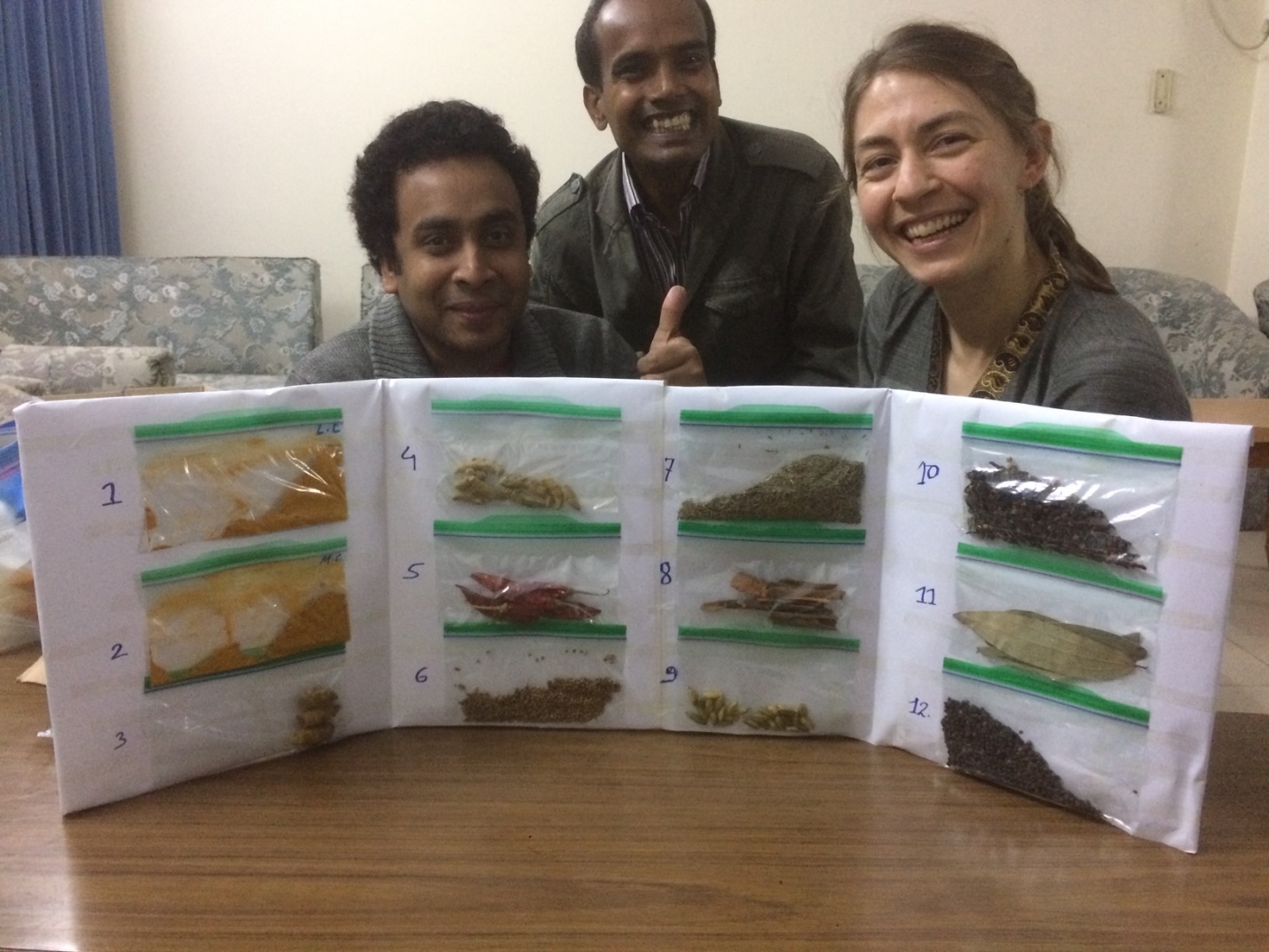


**Figure S1.** Spice board used during focus group discussions to elicit information regarding household spice usage and concerns about adulteration of spices.

# **S1-2. Lead-soldered cans**

| 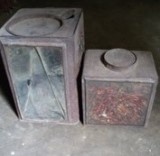 |
| --- |

**Figure S2.** Photo of lead-soldered cans used to store dry foods.

**Table S1.** Summary of in-depth interview responses about lead-soldered can usage.

| **Response** | | **Number of Households** | |
| --- | --- | --- | --- |
| Number of lead-soldered cans per household | 1 | | 4 |
|  | 2 | | 6 |
|  | 3 or more | | 10 |
|  | 5-9 | | 5 |
| Years using the lead-soldered can(s) | 10-14 | | 2 |
|  | 15-19 | | 5 |
|  | >20 | | 8 |
| Purchaser of the lead-soldered cans | Male head of household (husband, father, father-in-law) | | 15 |
|  | Mother-in-law | | 1 |
|  | None (gift from parental household for marriage)  *"I didn't purchase this* can*. After getting my marriage, my father gave me this* can *20 years ago. I have no idea about the price of this can even where this* can*comes from my parent house. I have been using this* can *since last 20 years and repaired it in two consecutive years in 2007 and 2013 by the local repairer (hawker). The repaired cost of this can was BDT 35.”* | | 4 |
| Foods stored in lead-soldered cans | Rice (Puffed, uncooked, boiled, flattened, ground) | | 20 |
|  | Turmeric | | 4 |
|  | Chili pepper | | 1 |
|  | Biscuits, cake | | 2 |
| Benefits of lead-soldered can usage | Protects foods from rats, cats, and insects due to the lid. | | 20 |
|  | Keeps food crispy and fresh in a moist climate | | 17 |
|  | Durability | | 20 |
|  | Affordable price | | 20 |
|  | Availability | | 20 |
| Disadvantages of using lead-soldered cans | Affected by rust (locally called *morichadhora*) | | 7 |
| Benefits of lead-free plastic containers | Durability | | 20 |
|  | Affordable price | | 20 |
|  | Availability | | 20 |
|  | Attractive with many colors and sizes | | 20 |
| Disadvantages of using lead-free plastic containers | None known | | 16 |
|  | Plastic smell (if stored >12-15 days) | | 2 |
|  | Poor protection from rats and insects | | 2 |

# **S1-3. Geophagy**


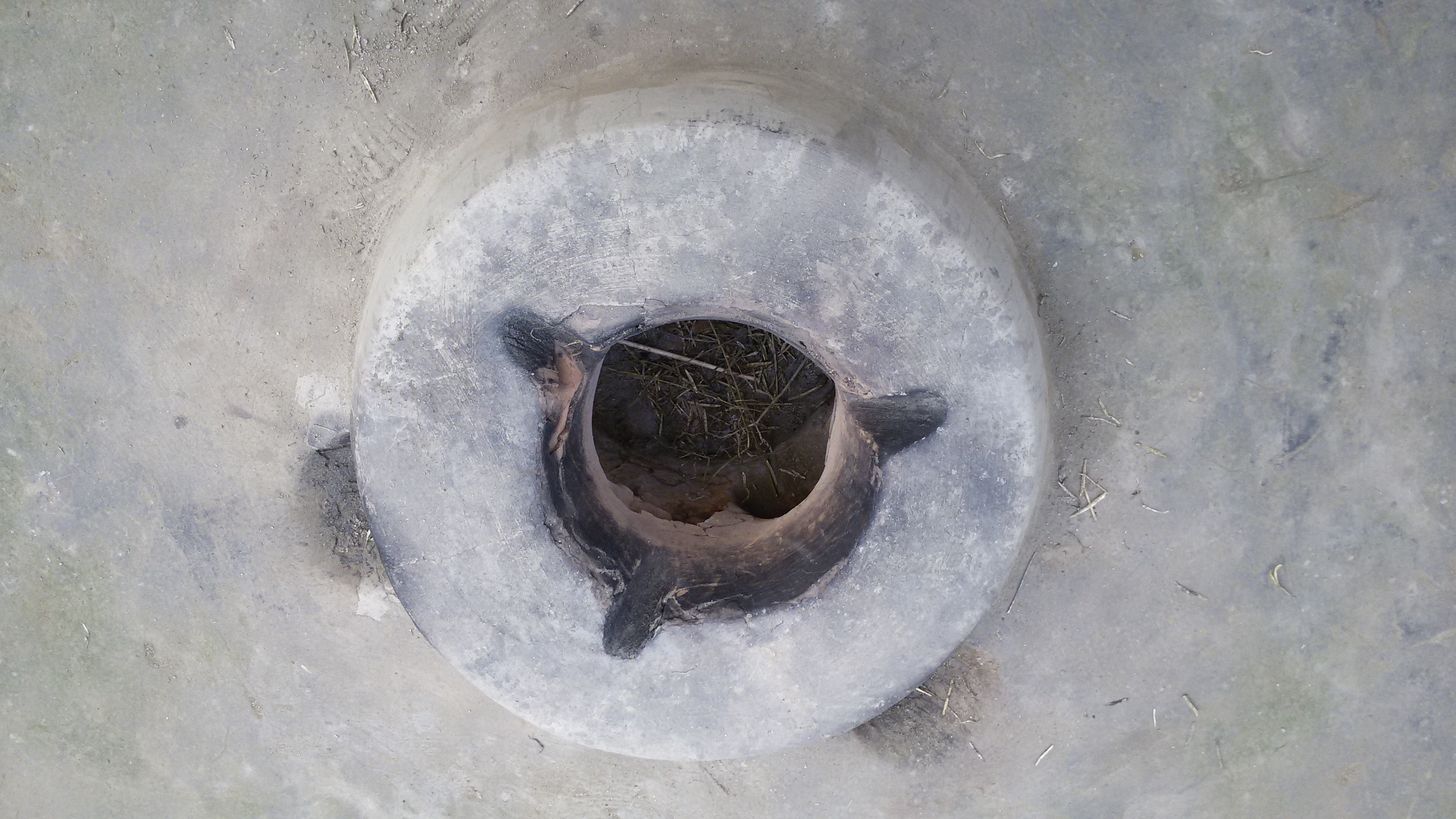


**Figure S3.** A stove (*chula*) where woman collect ash for consumption during pregnancy.

**Table S2.** Summary of in-depth interviews about geophagy.

| **Topic** | **Examples** | |  |
| --- | --- | --- | --- |
| Personal consumption of soil/clay | | • I consumed a fingerful amount of red soil from the burner (chula made of mud/clay) 3-5 times daily during my pregnancy. (R-1, 10) • I consumed small pieces of clay pot, clay toy 2-3 times daily during my pregnancy. (R-1, 10) • I used to consume *tirhi* (clay), bought from local market, 2-3 times daily during my pregnancy due to smell. (R-1, 8, 10)  • I ate *sikkamati* (red soil) twice during my whole pregnancy as it tasted good to me. The taste of the soil was sweet. (R-17, 20)  • I ate a fingerful amount of small pieces of clay pot three times in a day during my pregnancy because it tasted good to me. I felt the interest of eating it in my mind. I ate this during three of my pregnancy periods. I used to eat clay pot mostly after having meal and at noon or in the afternoon. I used to eat small piece (1-2 bites at a time) of clay pot 1-2 times in a week. I ate those clay pots in 7/8 months of the pregnancy periods. The clay pots were purchased from clay makers’ house. The smell of the clay pot was good which motivated me to eat. None suggested me to eat; I was self-motivated. (R-18) • I didn’t consume any soil/ soil tablets (baked clay) during my pregnancy. (R-2, 3, 4, 5, 6, 7, 9, 11, 13, 14, 15, 16, 17, 19, 20) | |
| Others' consumption of soil/clay | | • When I was young (18-30 years ago), I saw that some women consumed small pieces of clay pot during pregnancy. (R-1, 3, 4, 7, 10) • I saw in my childhood (3-20 years ago) that some neighboring women regularly took red soil from the burner during their pregnancy. I think that they like it for consuming so that they consumed it, nothing else. Now I don’t see it. (R- 2, 3, 4, 5, 6, 10) • I saw in my childhood that some women (e.g., elder sister, sister-in-law, mother, aunty, neighboring women) used to consume the *tirhi* during their pregnancy for seven to eight months. During pregnancy period, anytime anyone can take it if she wishes to get. I don’t know why. (R-5, 6, 7, 8, 10, R-11, 14, 15, 16)  • Sometimes I saw that some women consumed a fingerful amount of ash of cow-dung from their burner (locally name: *ghoborer chai*). They consumed it due to smell. I didn’t consume this during my pregnancy. (R-3)  • I saw some women in our area eat sikkamati but my mother-in-law suggested me not to eat this soil because it might cause harm to my body. (R-17, 20) | |

**S2. List of messages to influence the consumption of lead-contaminated goods at the household-level**

*The following focuses on messages to influence factors that affect behavior at the household level. Those that are underlinedwere deemed most salient and were incorporated into the behavior change communication plan (S3 below).*

**S2-1. Turmeric messages and associated theories**

The following are messages to raise awareness and influence factors that affect behaviors related to turmeric. Increasing self-efficacy has been omitted from this analysis since it is changed by learning from others and by doing oneself not via messages. RINEW focuses on messages to influence behaviors due to time and resource constraints associated with an integrated intervention. If time allowed, four ways to increase self-efficacy could be considered, as defined by Albert Bandura in his writing on Social Cognitive Theory and self-efficacy :i) Modeling (see someone doing it); ii) Demonstration (showing steps) - both modeling and demonstration you have vicarious experiences; iii) Practice (getting good at it); and iv) Repetition (doing it over and over). We can explore the extent to which increasing self-efficacy would improve the odds that women switch from powdered to unpolished dried (or raw turmeric root).

Here are messages that target individuals who cook with or purchase turmeric:

1. Perceived **susceptibility** to lead exposure through turmeric powder (relates to risk) (from the Health Belief Model (HBM) but not found to be as important as benefits or barriers in studies)

- We have found high levels of lead in the turmeric powder in this area, therefore if you eat turmeric powder you get lead that affects your brain.
- The people of this area add lead to turmeric.

1. Perceived **severity** of consequences of lead exposure through turmeric powder (from HBM but not found to be as important as benefits or barriers in studies)

- Consuming lead damages the brain.
- Lead will make you and your children stupid, unable to complete school, unable to hold a good job, and ultimately won't have a good life.
- It's terrible to think that the food I cook is poisoning my family.
- It's terrible to think that I'm buying powdered turmeric that is unpure and isn't really powdered turmeric.

1. Perceived **benefits** of lead exposure through turmeric powder (from HBM and found to be most important in studies)

- If you use unpolished dried turmeric (or raw turmeric), you get most value for your money. The taste is better and the color is better. Some grinders cheat you by adding chickpea flour to the powdered turmeric. Unpolished dried turmeric is pure so if you have it ground yourself or if you paste your own raw turmeric, you will get the best curries.
- Using unpolished dried turmeric will make your child smarter.

1. Perceived **barriers** of lead exposure through turmeric powder (from HBM and found to be most important in studies)

- We recognize that paste from raw turmeric is not storable so it would need to be pasted daily. Instead, purchasing unpolished dried turmeric and grinding in may be easier for storage.
- Unpolished dried turmeric may be less available in your area, but if you coordinate with women in this group you could organize to have one person purchase and grind turmeric for the group.

1. Cues to action to avoid turmeric powder (from HBM) - not so relevant here.

From other models:

1. Descriptive norms (Focus Theory of Normative Conduct of Cialdini, Reno and Kallgreen<https://en.wikipedia.org/wiki/Norm_(social)#Focus_theory_of_normative_conduct>)

- More and more people in your area are buying unpolished dried turmeric roots and grinding themselves.

1. Injunctive norms (Focus Theory of Normative Conduct of Cialdini, Reno and Kallgreen<https://en.wikipedia.org/wiki/Norm_(social)#Focus_theory_of_normative_conduct>)

- Your family doesn’t want you to use powdered turmeric since it is not pure and could poison them.
- Your mother-in-law doesn’t want you to use powdered turmeric since it is not pure and may not produce bright yellow-colored curries.

1. Nurture:

- You show love for my family by feeding them pure foods and avoiding powdered turmeric.

1. Disgust: not applicable
2. Scientific efficacy/Empirical efficacy : Although individuals are unable to observe the adverse effects of lead exposure, these concepts are difficult to include.

**S2-2. Lead-soldered cans**

Here are some messages about lead-soldered cans

1. Perceived **susceptibility** to lead exposure through cans

- We found lead-soldered cans in this area that are being used to store dried foods like puffed rice.

1. Perceived **severity** of consequences (from HBM but not found to be as important as benefits or barriers in studies)

- Consuming lead damages the brain
- Lead will make you and your children stupid, unable to complete school, unable to hold a good job, and ultimately won't have a good life

1. Perceived **benefits** (from HBM and found to be most important in studies)

- Lead-soldered metal cans become affected with junk *(locally called; morichadhora)* easily which comes off into the food whereas plastic containers don’t.
- Using plastic containers is more affordable. Metal cans are as much as 8 times more expensive than plastic containers. Metal cans need to be repaired over time but plastic containers do not.

1. Perceived **barriers** (from HBM and found to be most important in studies).
   - Plastic containers can give off a bad smell if food is stored in these types of containers for more than 12-15 days. This can be avoided by using good quality plastic containers.
   - Metal cans are often passed down in a family for decades as heirlooms or sometimes marriage gifts so they can have familial significance.
   - Metal cans keep puffed rice crispy even in humid climates. You could heat the puffed rice again or store smaller quantities at a time to ensure the puffed rice stays fresh. Otherwise, glass or unglazed clay pots will keep dry food fresh.
   - Some people think rats and insects destroy food in plastic containers easier than metal cans but this is rare if you keep the lid on tight and store in a location away from the ground. You should be able to avoid this. Otherwise, you could try glass or unglazed clay pots.
2. Cues to action to avoid lead-soldered cans (from HBM) - not so relevant here.

From other models:

1. Descriptive norms:

- The use of metal *cans* is decreasing day by day in your area due to the popularity of plastic containers and their availability in local bazaars and even door-to-door salesmen.

1. Injunctive norms:

- Your family doesn’t want you to store food in lead-soldered cans because the lead comes off the can into the food and poisons them.

1. Nurture:

- You show love for my family by feeding them foods from plastic containers that have no lead.

1. Disgust:

- It's terrible to think that the food you serve could poison your family.

1. Scientific efficacy/Empirical efficacy – not so relevant here.

**S2-3. Geophagy**

Here are some messages about lead-soldered cans

1. Perceived **susceptibility** to lead exposure through cans

- We found high levels of lead in clay and soil consumed by pregnant women and children in this area.

1. Perceived **severity** of consequences (from HBM but not found to be as important as benefits or barriers in studies)

- Consuming lead damages the brain.
- Lead will make you and your children stupid, unable to complete school, unable to hold a good job, and ultimately won't have a good life.

1. Perceived **benefits** (from HBM and found to be most important in studies)

- Lemon leaves and ginger make you better but soil and clay harm you.
- We are humans so we should consume food.

1. Perceived **barriers** (from HBM and found to be most important in studies)
   - Soil and clay smells good which attracts women to eat it.
   - Some people have religious misperceptions that since we all came from soil, there is no harm in eating soil.
   - Soil and clay has a unique earthy taste that other foods do not have.
   - It is good for children to play in the dirt.
   - It is difficult to control where children play and what they put in their mouth.
2. Cues to action to avoid soil or clay consumption (from HBM) - not so relevant here.

From other models:

1. Descriptive norms:

- The consumption of soil and clay among pregnant women is decreasing as women are understanding how harmful it is.

1. Injunctive norms:

- Your mother and mother-in-law do not approve of eating soil and clay.

1. Nurture:

- By taking care of yourself when you are pregnant, you are showing love for your family.

1. Disgust:

- It's terrible to think that I’m eating something as unclean as dirt.

1. Scientific efficacy/Empirical efficacy – not so relevant here.

**S3. Household-level Behavior Change Communication Plan: Messages and Recommendations**

[KEY:

Blue highlight = Behavioral recommendation*;*

Yellow highlight = Messages influencing behavioral factors]

Lead is a kind of metal. It is invisible. But, it is dangerous for our health. It is dangerous for the brains of young children. It can harm their brains very much. It can also harm us. Consuming lead will make you and your children less smart, less able to complete school, and less able to hold a good job. Lead makes it hard to have a good life. (Perceived severity)

We tested the blood of people living in Bangladeshi villages. We found that many of them had lead in their blood. We became very worried. We asked ourselves: Where is this lead coming from?

So, we tested soil, food and other things here and there in some Bangladeshi villages. We found that there are four common ways that lead can enter the body of a person.

The first way is through a yellow coloring powder that some people add to turmeric to make it more yellow. The vendors of turmeric think that people will not buy their turmeric powder, unless it is bright yellow. So the people who polish turmeric add a yellow coloring powder to make the turmeric bright yellow. But this yellow coloring powder is full of lead. We have found high levels of lead in the turmeric powder in this area. (Perceived susceptibility)If you eat turmeric that contains this yellow coloring powder, lead will enter your body. And it is very dangerous for both children and adults to have lead inside their bodies.

To avoid eating lead when you eat turmeric, you can buy the raw turmeric root or unpolished dried turmeric root, and paste or grind it yourself, instead of buying powdered turmeric.

- If you use unpolished dried turmeric (or raw turmeric), you get most value for your money. The taste is better and the color is better. Some grinders cheat you by adding chickpea flour to the powdered turmeric. Unpolished dried turmeric is pure so if you have it ground yourself or if you paste your own raw turmeric, you will get the best curries. (Perceived benefits)
- Unpolished dried turmeric may be less available in your area, but if you coordinate with women in this group you could organize to have one person purchase and grind turmeric for the group. (Perceived barriers)

So one way that people eat lead is by eating powdered turmeric that has lead coloring powder inside it.

But there is another way. Some people use a type of solder to repair old metal cans. These metal cans are big and strong. People like to store their food inside them. Unfortunately, the solder used to repair the old metal cans contains a large amount of lead. If you store food in these metal cans, the lead from the solder will enter your food. When you eat that food later, lead will enter your blood. And this is very dangerous. We found that lead-soldered cans in this area are being used to store dried foods like puffed rice. (Perceived susceptibility)

To avoid eating lead when you eat food stored in metal cans, you can store your food in plastic containers. You should never store your food in metal cans, especially old metal cans that have been repaired with solder.

- Plastic containers are better than lead-soldered metal cans:
  - Lead-soldered metal cans become affected with junk *(locally called; morichadhora)* easily which comes off into the food whereas plastic containers don’t. (Perceived benefits)
  - Also, using plastic containers is more affordable. Metal cans are as much as 8 times more expensive than plastic containers. Metal cans need to be repaired over time but plastic containers do not. (Perceived benefits)
- Because of the popularity of plastic containers and their availability at local bazaars and even from door-to-door salesmen, the use of lead-soldered metal cans is decreasing. (Descriptive norms)

So now we have talked about three ways you might be eating lead. Remember, you show love for your family by feeding them pure foods so don’t feed them poisonous lead from powdered turmeric, yellow-colored pitha, and food from lead-soldered cans. (Nurture)

There is final way we found that people eat lead. This is through eating soil or clay. Sometimes pregnant women crave soil or clay because they don’t feel well due to pregnancy. Children may also eat soil or chew on clay toys while playing. We found high levels of lead in the soil and clay of this area. (Perceived susceptibility)

Soil contains a lot of lead and therefore should not be eaten. Instead of eating soil or clay when you feel unwell during pregnancy, try smelling lemon leaves or eating ginger. Watch your young children to make sure they are not eating soil and clay.

- We are humans so we should consume food and not soil. Lemon leaves and ginger make you better but soil and clay harm you. (Perceived benefits)
  - Soil and clay smells good which attracts women to eat it without realizing what they are doing. We have to be aware of what we are putting in our bodies even though it may be difficult. (Perceived barriers)
  - The consumption of soil and clay among pregnant women is decreasing as women are understanding how harmful it is. (Descriptive norms)

So there are four things you can do to avoid lead. First, you can buy the raw or unpolished dried turmeric root, and paste or grind it yourself, instead of buying powdered turmeric. Second, you can eat uncolored pitha. If you need to eat yellow-colored pitha, prepare them yourself using raw or unpolished dried turmeric to add natural yellow color. Third, you can store your food in plastic containers instead of in metal cans. Fourth, you can use lemon leaves or ginger instead of eating soil or clay when you feel nauseous during pregnancy. And you can watch to make sure your young children are not eating soil and clay.

**Table S3.** Recommendations for change at different levels to reduce lead exposure.

| **Category** | **Target Level** | **Recommendations** | **Actions and corresponding theoretical constructs to influence behavioral factors** | **Corresponding Theory of Change or Model** |
| --- | --- | --- | --- | --- |
| Any | Government | Implement, monitor, and enforce policies to disincentivize the use of lead-tainted products | 1. Strengthen governmental action and regulatory capacity in the context of a weak state | 1. Policy-driven change |
| Turmeric | Polishers | Polishers should stop adding lead coloring compounds to the dried roots during polishing | 1. Increase perceived susceptibility and perceived severity of the polishers to lead poisoning from their own contact with it during processing turmeric 2. Increase perceived benefits of using turmeric powder instead of lead chromate colorants 3. Inspect and fine polishing mills with evidence of lead coloring compounds 4. Subsidize the production of or import of a cheap food-grade lead-free yellow coloring compound | 1. Behavior-based change – Health Belief Model 2. Behavior-based change – Health Belief Model 3. Economic disincentive-based change 4. Economic subsidy-based change |
|  | Wholesalers | Wholesalers should stop selling turmeric adulterated with lead chromate | 1. Increase perceived susceptibility and perceived severity among wholesalers to lead poisoning from contact with and consuming the turmeric they sell 2. Inspect and fine wholesalers found selling lead-chromate contaminated turmeric 3. Monitor for lead chromate and inform the public about wholesalers selling adulterated turmeric in order to put pressure on wholesalers 4. Engage the press and public to put direct pressure on adulterers | 1. Behavior-based change – Health Belief Model 2. Economic disincentive-based change 3. Economic demand-based change 4. Economic informal regulation-based change |
|  | Household - general | Buy raw turmeric root or unpolished dried turmeric root, and paste or grind it oneself, instead of buying powdered turmeric | 1. Increase perceived susceptibility and perceived severity of lead poisoning from turmeric among consumers 2. Increase self-efficacy to cook with raw or unpolished turmeric via demonstrations 3. Reduce perceived barriers to use of unpolished dried turmeric root by reducing travel time or inconvenience by (for example) coordinating groups of individuals to buy unpolished dried turmeric root and grind in a batch for more than one family | 1. Behavior-based change – Health Belief Model 2. Behavior-based change – Health Belief Model 3. Behavior-based change – Health Belief Model |
| Lead-soldered cans | Can repairers | Can repairers should not use lead solder | 1. Increase perceived susceptibility and severity among can repairers about lead poisoning and their exposure | 1. Behavior-based change – Health Belief Model |
|  | Household - general | Use plastic, glass, or unglazed clay containers instead of metal lead-soldered cans for dried food storage | 1. Increase perceived susceptibility and severity among family members about the harmful effects of lead 2. Create a program where individuals can sell back or exchange lead-soldered metal cans for plastic, glass or unglazed clay containers | 1. Behavior-based change – Health Belief Model 2. Economic incentive-based change |
| Geophagy | Household - Pregnant Women | Smell lemon leaves or eat ginger when feeling nauseous instead of soil or clay. | 1. Increase perceived susceptibility and severity among pregnant women about the harmful effects of lead and lead-containing soil and clay 2. Reduce perceived barriers by forming a supportive group of women to help each other overcome nausea, vomiting, and cravings during pregnancy | 1. Behavior-based change – Health Belief Model 2. Behavior-based change – Health Belief Model |
|  | Household - Children | Mothers or other family members should watch children so they do not eat soil or clay toys. | 1. Increase perceived susceptibility and severity among family members about the harmful effects of lead and lead-containing soil and clay 2. Increase mothers’ self-efficacy to prevent children from ingesting soil by demonstrating how to make safe play areas free from soil | 1. Behavior-based change – Health Belief Model 2. Behavior-based change – Health Belief Model |

**S4. Results**

## **Table S4.** Maternal and child uptake of calcium- and iron-rich food groups within the past 24 hours.

| **Food Groups** | **Maternal** | | **Child** | |
| --- | --- | --- | --- | --- |
|  | Control  Freq (%)  n = 227 | Intervention  Freq (%)  n = 241 | Control  Freq (%)  n = 222 | Intervention  Freq (%)  n = 233 |
| **Leafy green vegetables**   1. Pumpkin leaves, mustard leaves, bean leaves, amaranth green, pigeon pea/motorshuti leaves, drumstick greens, fenugreek greens (Methi), spinach, sweet potato leaves, turnip greens, pui shak, lau shak, napa shak, kolmi shak, helencha shak, kolai shak, shaobui shak, dimali shak, bothua shak, or other dark green leafy vegetables | 81 (36%) | 84 (35%) | 23 (10%) | 45 (19%) |
| **Meat, poultry and eggs**   1. Liver, kidney, heart, or other organ meat | 6 (3%) | 2 (1%) | 8 (4%) | 5 (2%) |
| 1. Any type of flesh from birds and animals | 62 (27%) | 49 (20%) | 33 (15%) | 31 (13%) |
| 1. Any type of egg | 39 (17%) | 49 (20%) | 47 (21%) | 63 (27%) |
| **Fish and seafood**   1. Fresh fish, dried fish (shutki), crab (freshwater), or other fish or seafood | 191 (84%) | 218 (91%) | 85 (38%) | 133 (57%) |
| **Pulses, legumes, nuts, and seeds**   1. Chickpea, lentils, peas, peanuts, soybeans, soy products, almonds, cashews, pistachios, sesame seeds, sunflower seeds, pumpkin seeds, or peanut butter | 74 (33%) | 100 (42%) | 52 (23%) | 76 (33%) |
| **Dairy**   1. Cheese, yogurt, curd, ice cream, curdled milk or other foods made with milk | 7 (3%) | 7 (3%) | 15 (7%) | 8 (3%) |
| 1. Animal’s milk that is fresh, powdered, or tinned | 1 (1%) | 2 (1%) | 116 (52%) | 122 (52%) |

## **Table S5.** Intake of calcium- and iron-rich food groups within the past 24 hours.

| **# of food groups** | **Maternal** | | **Child** | |
| --- | --- | --- | --- | --- |
|  | Control  Freq (%)  (n=227) | Intervention  Freq (%)  (n=241) | Control  Freq (%)  (n=222) | Intervention  Freq (%)  (n=233) |
| 0 | 5 (2%) | 3 (1%) | 24 (11%) | 10 (4%) |
| 1 | 68 (30%) | 59 (25%) | 84 (38%) | 67 (29%) |
| 2 | 93 (41%) | 101 (42%) | 66 (30%) | 84 (36%) |
| 3 | 43 (19%) | 63 (26%) | 31 (14%) | 44 (19%) |
| 4 | 13 (6%) | 14 (6%) | 15 (7%) | 24 (10%) |
| 5 | 4 (2%) | 1 (1%) | 2 (1%) | 4 (2%) |
| 6 | 1 (1%) | - | - | - |
| 7 | - | - | - | - |
| 8 | - | - | - | - |
